# “Take the tablet or don’t take the tablet?”—A qualitative study of patients’ experiences of self-administering anti-cancer medications related to adherence and managing side effects

**DOI:** 10.1007/s00520-023-08122-6

**Published:** 2023-11-07

**Authors:** Thu Ha Dang, Clare O’Callaghan, Marliese Alexander, Kate Burbury, Prem Prakash Jayaraman, Nilmini Wickramasinghe, Penelope Schofield

**Affiliations:** 1 Department of Psychological Sciences, School of Health Sciences, Swinburne University of Technology, Melbourne, VIC Australia; 2 Department of Health Services Research and Implementation Science, Peter MacCallum Cancer Centre, Melbourne, VIC Australia; 3 Digital Health Cooperative Research Centre, Sydney, Australia; 4 Caritas Christi and Psychosocial Cancer Care, St Vincent’s Hospital, Melbourne, VIC Australia; 5 Department of Medicine, St Vincent’s Hospital, The University of Melbourne, Melbourne, VIC Australia; 6 Pharmacy Department, Peter MacCallum Cancer Centre, Melbourne, VIC Australia; 7 Sir Peter MacCallum Department of Oncology, The University of Melbourne, Melbourne, VIC Australia; 8 Digital and Healthcare Innovation, Peter McCallum Cancer Centre, Melbourne, VIC Australia; 9 Factory of the Future and Digital Innovation Lab, School of Science, Computing and Engineering Technologies, Swinburne University of Technology, Melbourne, VIC Australia; 10 Optus Digital Health, La Trobe University, Melbourne, VIC Australia; 11 Department of Health and Bio Statistics, School of Health Sciences and Iverson Health Innovation Research Institute, Swinburne University of Technology, Melbourne, VIC Australia; 12 Epworth Healthcare, Victoria, Australia; 13 Department of Psychological Sciences and Iverson Health Innovation Research Institute, Swinburne University of Technology, Melbourne, VIC Australia; 14 Digital Cancer Care Innovation, Department of Health Services Research, Peter MacCallum Cancer Centre, Melbourne, Australia

**Keywords:** Medication adherence, Oral anti-cancer, Qualitative research, Side effects, Self-management

## Abstract

**Purpose:**

Medication non-adherence is a well-recognised problem in cancer care, negatively impacting health outcomes and healthcare resources. Patient-related factors influencing medication adherence (MA) are complicated and interrelated. There is a need for qualitative research to better understand their underlying interaction processes and patients’ needs to facilitate the development of effective patient-tailored complex interventions. This study aimed to explore experiences, perceptions, and needs relating to MA and side effect management of patients who are self-administering anti-cancer treatment.

**Methods:**

Semi-structured audio-recorded interviews with patients who have haematological cancer were conducted. A comparative, iterative, and predominantly inductive thematic analysis approach was employed.

**Results:**

Twenty-five patients from a specialist cancer hospital were interviewed. While self-administering cancer medications at home, patients’ motivation to adhere was affected by cancer-related physical reactions, fears, cancer literacy and beliefs, and healthcare professional (HCP) and informal support. Patients desired need for regular follow-ups from respectful, encouraging, informative, responsive, and consistent HCPs as part of routine care. Motivated patients can develop high adherence and side effect self-management over time, especially when being supported by HCPs and informal networks.

**Conclusion:**

Patients with cancer need varied support to medically adhere to and manage side effects at home. HCPs should adapt their practices to meet the patients’ expectations to further support them during treatment. We propose a multi-dimensional and technology- and theory-based intervention, which incorporates regular HCP consultations providing tailored education and support to facilitate and maintain patient MA and side effect self-management.

**Supplementary Information:**

The online version contains supplementary material available at 10.1007/s00520-023-08122-6.

## Introduction

Oral cancer therapies have contributed to significantly improved survival [1–3] and reduced hospital stays for patients when compared to traditional chemotherapy treatments [4]. However, to be effective, oral treatments require medication adherence (MA), meaning that the patient needs to take medications according to their doctor’s prescription [5] throughout long-term treatment [6, 7]. Despite the importance of MA, its rates varied between patients with different cancer types and could be as low as 14% for some anti-cancer regiments [8–10]. Consequently, patients may have low survival rates [11–13], disease progression, reduced functional ability, increased risk of hospitalisation, lower quality of life [3, 14–17], and increased utilisation of healthcare resources. Given the significant concern of medication non-adherence problems in cancer, there has been increasing research focus on its associated barriers [8, 18, 19] and solutions [5, 20–22]. Generally, MA is a complicated phenomenon that is influenced by multiple dimensions (groups of factors): socio-economic, health systems, and condition-, therapy-, and patient-related [20, 23]. Patient-related group of factors, including cognitive, psychological, and interpersonal, is most important due to MA interventions potentially making the most impact on this group [23, 24]. However, these factors vary between patients and interact with idiosyncratic illness challenges to influence adherence behaviour in ways not yet fully understood [23, 25]. Thus, there is a need for qualitative research to understand the underlying processes influencing medication non-adherence among patients with cancer and their needs, in order to facilitate the development of patient-tailored complex interventions [26]. This qualitative study aimed to explore experiences, perceptions, and needs relating to MA and side effect management of patients who are self-administering anti-cancer treatment.

### Research context

We developed a beta version of the Safety and Adherence to Medication and Self-care advice in ONcology mobile application (SAMSON mobile app) to support people with cancer’s adherence to their oral treatment. After development, we tested the app on people with haematological cancer at an Australian metropolitan oncology hospital. SAMSON mobile app testing was a mixed-method study aiming to (a) assess the quality of a SAMSON mobile app to ensure it is fit for purpose and user-friendly and provides appropriate supporting MA information and (b) obtain data regarding the patient’s experience, expectation, and perception of the app. Participants used the app for 6 weeks. After that, they were invited to complete quantitative questionnaires on the app’s evaluation and to attend a qualitative interview. Participants could choose to participate solely in the quantitative part, or in both quantitative and qualitative parts of the SAMSON mobile app testing study. The qualitative study presented here is part of the SAMSON mobile app testing.

## Methods

### Design

The study was informed by the constructivist paradigm which asserts that reality perception is constructed from our individual, social, and historical contexts, rendering the existence of no absolute shared truth [27]. Qualitative thematic analysis [28, 29] was conducted using selected techniques derived from grounded theory, including comparative, iterative, and predominantly inductive analysis [30]. One of the creators of grounded theory, Anselm Strauss, asserted that researchers could also use selected grounded theory techniques to conduct thematic analysis [30].

### Setting and participants

Participants were recruited through the Haematology Outpatient Clinic at Peter MacCallum Cancer Centre in Melbourne between September and December 2021 as part of the SAMSON mobile app testing. During data analysis, several themes were generated that related to how patients were experiencing the management of MA and accompanying side effects. These findings are reported in this paper.

Inclusion criteria were as follows: (1) had an established diagnosis of chronic lymphocytic leukaemia (CLL), chronic myeloid leukaemia (CML), essential thrombocythaemia (ET), myelofibrosis, myeloproliferative neoplasms, or polycythaemia (Rubra) vera; (2) currently treated with or about to commence oral treatment for their disease; and (3) over 18 years old. Patients were excluded from the study if (1) they were too unwell to participate as determined by the treatment team or (2) the remaining time indicated for the treatment was less than 6 weeks.

Using convenience sampling, patients were identified, informed, and screened by their caring physicians when attending outpatient clinic appointments. Eligible patients were referred to the research coordinator (THD) to go through a comprehensive informed consent process.

### Interview/data collection

Consenting participants were scheduled for an interview within 6 to 8 weeks after the study’s commencement. Interviews were conducted either face-to-face at the clinic or online via Zoom, between November 2021 and February 2022, by THD who was trained in qualitative research. A semi-structured interview guide, developed and reviewed by experts in qualitative research methodology, psychology, and digital health, was employed (see Appendix 1). Interviews were audio recorded and then anonymously transcribed verbatim [31].

### Data analysis

Data analysis included a qualitative inter-rating process to strengthen the study’s credibility and trustworthiness [32–34]. Firstly, THD coded all interview records with descriptive labels that represented text segments. Secondly, CO, an experienced qualitative researcher, reviewed all interviews and THD’s codes and agreed or disagreed or suggested additional codes. Both researchers then discussed the codes for the entire data set until reaching an agreement. Codes were then collated and labelled into sub-categories (representing comparable code groups), categories (representing comparable sub-category groups), and themes (representing comparable category groups). THD led category and thematic development, and CO reviewed these. Adjustments were made until both analysts agreed on the final representation of the findings. QSR NVivo version 12 qualitative data management software [35, 36] was used to support data management during the analysis. Study reporting was guided by the Consolidated Criteria for Reporting Qualitative Research (COREQ) [37].

## Results

### Characteristics of the population

Among 30 patients who participated in the SAMSON mobile app testing, 25 (18 male) were interviewed. Mean age was 58 (range 30–74) years. Most patients had CLL or CML and were treated with varied oral therapeutics. Average time since diagnosis was 7.2 years (SD = 6.7). Average interview duration was 41 (range 19–98) min. Characteristics of participants are presented in Table 1. Three themes were generated and are described below. A full presentation of themes, categories, and sub-categories is in Appendix 2.

**Table 1:** General characteristics of the participants

Participants (*n*)	25
Male, *n* (%)	18 (72)
Age (years), mean (SD)	57.59 (12.47)
Diagnoses, *n* (%)	
Chronic lymphocytic leukaemia	14 (56)
Chronic myeloid leukaemia (CML)	10 (40)
Essential thrombocythaemia (ET)	1 (4)
Treatment, *n* (%)^a^	
Acalabrutinib	1 (4)
Dasatinib	4 (16)
Hydroxyurea	1 (4)
Ibrutinib	2 (8)
Imatinib	6 (24)
Venetoclax	11 (44)
Participants were about to commence oral treatment, *n* (%)	4 (16)
Time since diagnosis (years), mean (SD)	7.19 (6.65)
Length of interview (minutes), mean (range)	41 (19–98)

^a^All participants were on one oral anti-cancer medicine

### Theme 1: Varied factors affect patient motivation of MA

#### Cancer-related physical reactions and fears

Most patients experienced cognitive and psychological distress when managing their diagnosis and adhering to treatment. The diagnosis was described as “frightening” (P10, 63-year-old female, diagnosed with CML) and “scary” (P12, 57-year-old female, diagnosed with CLL), and disease-related social isolation gave P12 “a lot of tears in the heart”. Many patients knew that the “horrible” (P05, 67-year-old female, diagnosed with CLL) self-administered cancer treatment would cause side effects; however, all chose to adhere because the disease negatively affected much of their life and well-being. P23 (62-year-old male, diagnosed with CLL) said, “these lumps do get big” and “I can’t sleep, and I can’t rest with them”. Nonetheless, some patients who did not have many disease-related symptoms and were “feeling okay” (P12) could doubt the need for therapy. While no patient chose to not adhere to cancer treatment because of side effects, P18 (60-year-old male, diagnosed with ET) reported that others may not adhere if they thought that treatment benefits did not outweigh the side effects. P18 explained, “even though their [patients’] life might be extended by a short period of time, the quality of life is not there. So, they’ve [patients have] chosen to go with no treatment”.

#### Cancer literacy and beliefs

Generally, cancer literacy and confident beliefs about cancer treatment motivated patients, with P18 stating, “(I) take these drugs to keep me going”, and that “adhering to medication is ‘a no brainer’”. Most patients were aware that, cancer is a “serious condition” (P27, 56-year-old female, diagnosed with CLL) that needs “100% (adherence)” to “get a good result” (P06, 61-year-old male, diagnosed with CLL). They understood that if they did not take the medicine regularly, their cancer “would have come back” and then their “life span would have been dramatically cut short” (P18). Patients’ confidence and belief in treatment increased when it worked, and their condition improved. P14 (65-year-old female, diagnosed with CLL), for example, felt “encouraged” when having “no bone pain at all”.

Some patients believed that being positive and determined were helpful in MA and treatment outcomes: “If you’ve got the right thought to it and you don’t expect things to occur, they won’t… I’m not going to go looking for side-effects” (P09, 43-year-old male, diagnosed with CML). P18 also believed that “the attitude has a lot to do with it [disease outcome]. If you’re a positive person you’re more likely to have a positive outcome”. In addition, religious beliefs could also motivate some patients: “God is there to help me” (P12).

#### HCP communication

Patients’ motivation to comply with the treatment was influenced by their trust in HCPs and healthcare services and related support. P20 (48-year-old male, diagnosed with CML) commented, “just stick with what your doctor tells you and yeah, hopefully everything works out”. P12 acknowledged, “I am lucky this hospital is the best hospital; I have the best doctor”.

HCP’s poor communication skills, however, could reduce patients’ trust in healthcare and instil doubts about MA. A patient revealed their experience when communicating with an HCP:

she [nurse] makes me feel very anxious as well and she ignored my phone… she caused me confusion about a lot of things. I requested clarification, she don’t want to reply, and she kept me waiting, I don’t know, take the tablet, or don’t take the tablet? (P12)

#### Family and friends’ support networks can help with disease management and MA

Living with supportive families especially helped patients to medically adhere: “Family support is a great thing “, P20 said. When adjusting to taking his medications “in the early days”, P06 said, “My wife used to just say, ‘Have you taken your tablets?’”, and P29 (72-year-old male, diagnosed with CLL) added, “It’s good that you’ve got someone there to give you a kick up the backside and say, ‘Come on, take your tablets’”. P01 (46-year-old male, diagnosed with CLL) said it was a “shame” that those without family encouragement would struggle with treatment. Support from friends was also described as “very important” (P24, 55-year-old male, diagnosed with CML) in patients’ management of their disease and cancer therapy.

### Theme 2: When motivated, MA and side effect management strategies can develop over time

#### Developing personal strategies to support MA

Most patients had been on oral anti-cancer treatments for many years, with two describing medication intakes as “routine” (P18) and a “daily thing” (P24). This habitual and routine behaviour could have commenced during initial cancer treatment hospitalisation when, for example, P08 (57-year-old male, diagnosed with CLL) was told to “have it [the medicine] within half an hour of breakfast”, and P30 (66-year-old male, diagnosed with CLL) was given a “starter box… (that) just laid out the right number of tablets on the right day…in the first 4 or 5 weeks”. Many others, however, only developed routine MA behaviour over time while discovering and applying personal mnemonic strategies. These could be physical reminders, such as putting “all drugs into a weekly box of tablets” and “leaving them on the fridge” (P10), or digital reminders, such as “phone prompt notifications”, proving the popularity of smartphones nowadays (P15, 37-year-old male, diagnosed with CML). Timing of medication intake could also be adjusted as desired, for example, P24 “chop(ped) and change(d)” medication intake timing to cope with daily life. P30 also said, “What I have been doing is putting off the taking of the venetoclax [treatment for haematological cancer] until around about 11 o’clock, until after I’m off the golf course”. Moreover, a few patients could establish personal ways to avoid overdosage, such as “I use a black Texta and on each of the tablets, I put a date…It’s a backup so that I don’t end up taking a double dose” (P10).

One patient also confirmed that having a strong mindset about the benefits and risks of taking medicine and developing strategies to manage side effects would be very important in MA.

If you’re not consistent with it [taking medications], your body can start not to respond to it… and then you’re potentially opening a whole lot of other problems for yourself. So that … even if the side-effects…were really bad, I probably wouldn’t have a choice…. The best way I can deal with that is just to come up with ways to manage the side-effects. (P16, 34-year-old female, diagnosed with CML).

Despite using MA strategies, two patients admitted that they had missed doses at “the beginning” (P24) or during the course of treatment “occasionally” (P23).

#### Managing side effects through various strategies

Medication side effects could be a challenge to adherence to many patients with cancer, as P29 commented, “When I first started ibrutinib [treatment for haematological cancer], it really knocked me around…for a while there it was really painful and sore”. To deal with medication side effects, patients shared many different strategies. They could seek and follow advice from HCPs or informal online sources, such as a “CML group of patients” (P16) and “Google” (P12). Many others tried to monitor and protect their bodies from the risks of medicines’ side effects by, for example, “always hav(ing) gloves on…not to use a razor to shave” to avoid bleeding (P29). A few used “trial and error” to avoid digestive issues. P15 could eat “prior to taking my tablets” to avoid nausea, and P10 tried to “have my tablet of an evening”, because the thought of “going to bed and lying down after having an evening meal and the tablet was making like a reflux”. Meanwhile, P24 preferred to take the pill “in the morning”, because if he got nauseous, he “would rather be at home than be at work dealing with that [nausea]”.

### Theme 3: Further HCP support needed while managing MA and side effects

#### Need for regular follow-ups from consistent HCPs to support MA

When adhering to medication treatment at home, patients wanted communication with HCPs as needed. Several, however, had not received any follow-up phone calls about their medications and some criticised the hospital’s suboptimal communication protocols. After reporting a “serious” medication side effect to their hospital contact person, P17 (63-year-old female, diagnosed with CLL) received an email stating that the contact was on holiday and to contact another person who responded “weeks” later. P12 also perceived inconsistent messaging on how medications would be ingested (inpatient, outpatient, or at home) and received “too much information altogether”. Some patients also wanted consistent HCP follow-up care, with P14 explaining, “it doesn’t help to have all these different people, you just need one person… too many people… just keep it simple because your head doesn’t work so well at the start part with the infusions.”

#### HCPs’ ongoing information, monitoring, and support could encourage MA

Patients regularly explained that ongoing connection with care teams would encourage MA. Individuals wanted HCP information about their idiosyncratic diseases, treatments, and potential medicines’ side effects, the importance of MA, how to overcome adherence barriers (if needed), and guidance on “(when) should you contact your doctors?” (P16) with concerns about health changes. Some also said that HCP follow-up would help them to feel valued and reassured, which was especially important for those, “terrified to make contact with medical people” (P5). They could also alert HCPs to patients needing additional support to medically adhere.

[HCPs] can ring and say “How are you going?”. It just makes you feel more important…You want to make sure that all are going along. Then if they are not complied [with the medication], you just say “I notice you haven’t been taking it. Is there a reason?” (P5)

Occasionally patients suggested how HCPs could encourage them with MA, including through providing “positive information” (P13, 68-year-old male, diagnosed with CLL) and “praise” (P5). P27 emphasised that conversations should not be “a blaming … but more encouraging and explaining”. P27 also advised that HCPs should, “as part of the routine care”, regularly ask specific questions about patient health or about the reason why they were non-adherent rather than general, open-ended enquiries. Patients suggested the point of contact could be a nurse (P5 and P10) or a pharmacist (P27).

Individuals recommended various time frames for follow-up calls, including “within the first 48 h” of commencing treatment, “then maybe 3 or 4 days after, and then a week later” (P24), once a week (P28, 42-year-old male, diagnosed with CML), or as determined by HCPs’ assessments of how patients are managing the medication (P23 and P27). Some also stressed the need for HCP information and discussions to maintain psycho-social well-being while adhering to cancer medications, including on whether their disease is genetic as P10 said, “Can I get pregnant?”.

Most patients also supported phone prompt MA reminders, along with HCP regular contact, as an option to promote MA; however, one patient commented, “I’m just not too sure how many people would enjoy a telephone call from a clinician every other day or whatever” (P30).

## Discussion

**Fig. 1:**
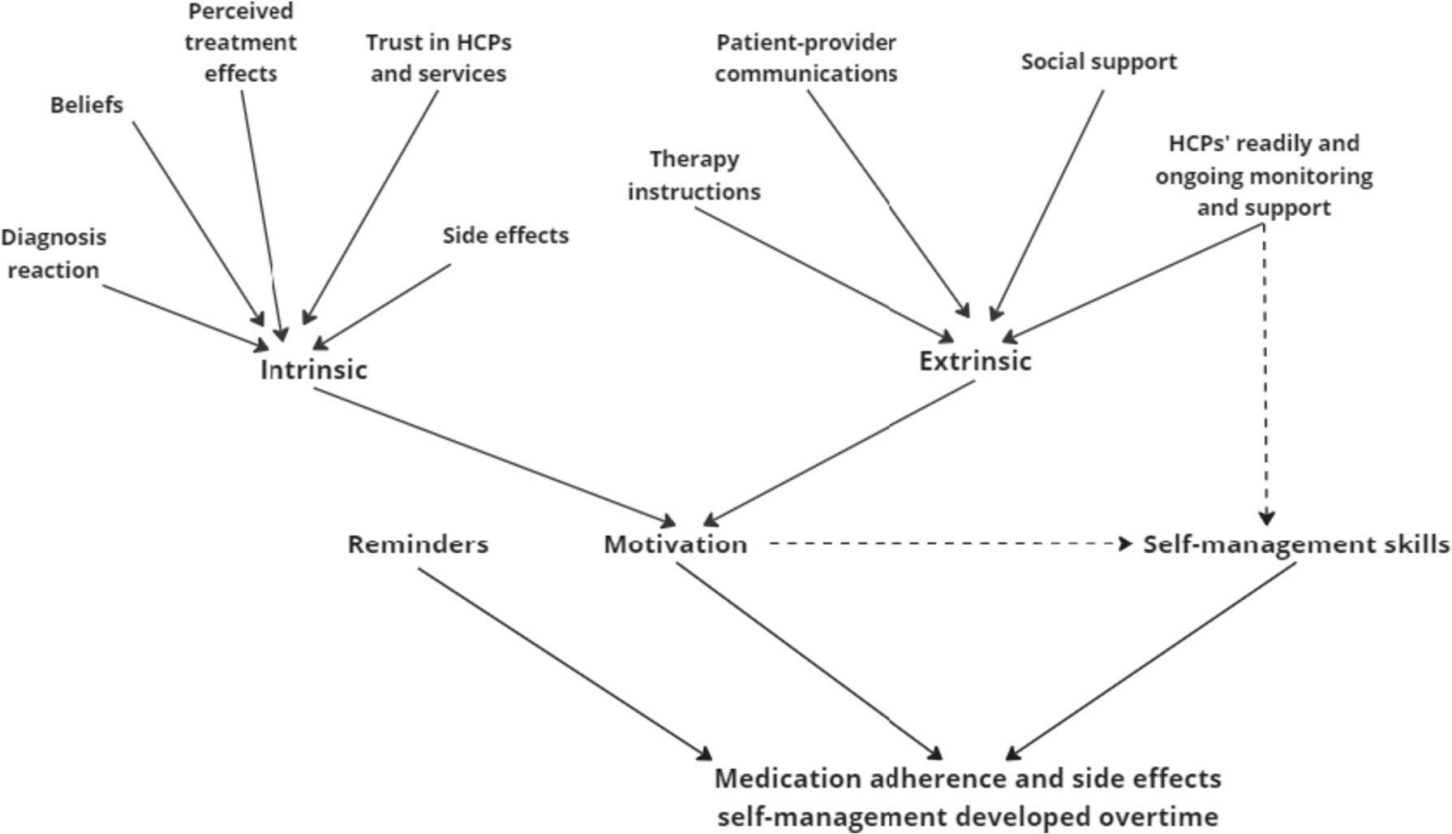
Medication adherence and side effect self-management strategy development

These results provide valuable insight into how patients’ motivation in MA is positively and negatively affected by various biopsychosocial factors. These can be grouped into two categories: intrinsic and extrinsic (Fig. 1). Intrinsic factors often motivate/de-motivate people to perform an activity for their own self-satisfaction, while extrinsic factors refer to people performing/not performing the activity to achieve certain outcomes [38].

In this cohort of patients with haematologic malignancies, intrinsic factors associated with MA included disease-related physical reactions and fears, cancer literacy, positive beliefs about treatment efficacy, and trust in HCPs and healthcare services. Conversely, perceptions that therapy is unnecessary or that treatment benefits are outweighed by harsh side effects could hinder patients’ motivation for treatment. This finding is consistent with the literature: MA behaviour is strongly influenced by patients’ beliefs, motivations, and perceptions of the disease and treatment [39–41]. Enabling MA factors, such as awareness of the need for medication and the importance of maintaining positive beliefs about the effectiveness of the treatment could raise the patients’ tolerance threshold to treatment side effects [42–44].

Extrinsic factors associated with disease management and MA in patients included support from family and friends, patient-provider communication including therapy instructions, and readily available and ongoing monitoring and support from HCPs. External support can help patients to overcome negative emotions post diagnosis and treatment commencement, and to feel more confident and motivated to manage their medical conditions and adherence. Previous studies in cancer and hypertension have also emphasised the importance of social and medical support in MA [44–46]. Both intrinsic and extrinsic factors contributed to the development of patients’ self-efficacy, i.e. MA and side effect management ability [47] and maintaining it over time [48].

The need for medical support from HCPs and ongoing follow-ups to promote MA and side effect self-management was a striking finding (Fig, 1). Participants desired regular connection with responsive and consistent HCPs and information on treatment rationales, side effects, and management. Other research has also shown that HCP support helps patients to develop self-management strategies for MA and medication side effects [49]. Study participants also desired regular follow-ups with communicative HCPs as part of the “routine care”. Participants reported that nurses or pharmacists could be the point of regular contact, which is in line with conclusions from previous studies on cancer [42, 44].

Medication side effects as a major barrier to MA has been discussed in recent literature in cancer care [44, 46, 49]. Most participants were willing to “deal with” the side effects and disclosed various personal strategies to cope with them. These findings echo recent qualitative findings [44] that side effects have a negative impact on MA motivation to oral chemotherapy; however, patients are willing to deal with them. This contrasts with some studies which found that people with non-malignant chronic illnesses may avoid side effects by not adhering to medication [7, 50, 51]. This difference could be due to the MA motivation facilitators related to a cancer diagnosis, in that a treatment’s potential benefits may be more likely to outweigh the barriers of unpleasant side effects when the treatment is for life-threatening cancer compared to a non-life-threatening chronic condition.

In this study, two patients confirmed that forgetfulness could be a challenge to MA, which is supported by previous research [9]. Patients reported using different reminder strategies: pill boxes, calendar reminder, or mobile app notifications to establish a medication-taking habit early on and throughout the course of treatment (Fig. 1). These strategies, especially the evolving use of digital technologies, to overcome this challenge have been reported previously [20].

### Clinical implications

Our study has illustrated that motivated patients can develop high adherence and side effect self-management over time, especially when being supported by HCPs and informal networks. MA motivation of patients with cancer can differ from those of other chronic diseases, which should be considered when introducing potential MA interventions. Our study reinforced previous findings reporting that MA is a complicated phenomenon influenced by multi-dimensional factors [23], and supports the perspective that multi-dimensional and multi-theory informed MA interventions will be most efficacious in this area [20, 52].

### Study limitations

This study was conducted at a single haematology department in an Australian specialist cancer hospital. As such, participants’ perceptions of MA and side effect management may not represent those who receive care elsewhere. Despite this, the findings improve the limited knowledge available about the real-life experiences of patients being treated with oral medicines for haematological cancers. The differences in participants’ disease situations and the lengths of time undergoing oral cancer treatments were not considered in this study. Since patients’ adherence behaviours can develop over time, MA motivation and status and self-management skills are likely to differ according to their illness and treatment history. Subsequent studies should consider grouping patients according to their cancer stage and length of time on treatment.

## Conclusions

This study illuminates the real-life experiences and perceptions of patients being treated with oral anti-cancer medicines and the diverse factors associated with how they can develop personal strategies for MA and side effect self-management. Varied supports are needed to help patients to medically adhere and manage side effects at home. These findings could help HCPs to consider how their practices can be adapted according to patients’ need for support during home treatment. It demonstrated that a multi-dimensional approach is needed to maintain and improve MA in cancer care, tailored to patients’ needs, and based on technology and multiple cognitive and behavioural theories. The approach would need to incorporate regular HCP consultations, to provide education and support to facilitate and maintain patients’ MA and side effect self-management.

### Supplementary Information

Below is the link to the electronic supplementary material.

Supplementary file1 (DOCX 28 KB)

Supplementary file2 (DOCX 20 KB)

## Data Availability

The data that support the findings of this study are available from the corresponding author upon reasonable request.

## Abbreviations

CML: Chronic myeloid leukaemia
CLL: Chronic lymphocytic leukaemia
ET: Essential thrombocythaemia
HCP: Healthcare professional
MA: Medication adherence
SAMSON: Safety and Adherence to Medication and Self-care advice in ONcology
