# Supplementary material for: “Take the tablet or don’t take the tablet?”—A qualitative study of patients’ experiences of self-administering anti-cancer medications related to adherence and managing side effects
Source: Support Care Cancer. 2023 Nov 7;31(12):680. doi: 10.1007/s00520-023-08122-6 (PMC10630231; doi:10.1007/s00520-023-08122-6)
Supplement: Supplementary file 1 — Supplementary file1 (DOCX 28 KB) [file 520_2023_8122_MOESM1_ESM.docx]

**Journal of Supportive Care in Cancer**

**Research**

“Take the tablet or don’t take the tablet?”–A qualitative study of patients’ experiences of self-administering anti-cancer medications related to adherence and managing side-effects

**Appendix 1. A beta-testing of SAMSON mobile health application (Safety and Adherence to Medications and Self-care advice in Oncology) on oncology patients.**

**INTERVIEW GUIDE**

Participant ID #: _____________________ Time: ____________________

| **INTRODUCTION & SUMMARISED INTERVIEW PROCEDURE** |
| --- |

1. Interviewer (research coordinator) introduces herself.
2. Interviewer explains the purpose of the interview is to to explore the needs of oncology patients regarding information and support to adhere to medication treatments and to manage issues relating to drug treatments in their homecare setting; and to provide opportunities for patient’s expectations, experience and perceptions of SAMSON.
3. Interviewer explains the interview procedure:

- Total interview time is about 30-40 minutes
- Interview will be recorded, then transcribed for further analysis
- All of patient’s information will be de-identified after the interview. This means that patient’s name will be replaced by a unique participant code, so that their personal identity is not connected with their data.
- Patient does not have to answer questions if they do not want to.
- Patient can stop the interview anytime if they do not want to participate any longer.

1. Interviewer informs patient that she will now start the interview and the audio/video tape will now be turned on.

| **NEEDS OF INFORMATION AND CARE SUPPORT** |
| --- |

1. **Treatments**
2. How long have you received oral cancer drug therapy for [*the name of cancer type*]?
3. **Disease and treatment experience**
4. In your opinion, how important is it for you to take your drugs as prescribed for your cancer? Why? Why not?
5. Are there times when you haven’t taken your medication as prescribed? *Can you tell me more about why you sometime don’t take your* medication?
6. Are there any drugs which you find harder to take as prescribed? Which ones? Why are these harder?
7. *(If patient adhered well to the treatment)* What strategies helped you most to remember to take your drugs daily?
8. What side-effects of your medication have you experienced?
9. What side-effects may make you reduce your medication dose or stop using your medicaiton?
10. What strategy did you use to limit these side-effects? What source of information did you find these strategies from?
11. **Expectation**
12. What do you think might be most helpful to you to support you to take your medication as prescribed?
13. Many people who have been diagnosed with cancer have questions and concerns about the medication they are prescribed. What questions or concerns relating to your medication would you like to ask?

| **EXPERIENCE, EXPECTATION AND PERCEPTION OF SAMSON** |
| --- |

**SAMSON mobile app**

1. How was your overall experience with SAMSON mobile app? (specifically, about:

- Medication reminder notifications
- Motivational messages that came with the weekly adherence rate
- Side-effects survey
- Ability to report serious side-effects
- Side-effects advice pages

1. What did you like about using the app?
2. What did you dislike about using the app?
3. If you were an app designer, what would you do to improve SAMSON? (see more questions in details)

| **INTERVIEW ENDING** |
| --- |

Interviewer asks patient if they have any questions or comments and thanks patient for their time that they have given to the study.

**More questions about the app in details (optional)**

1. Reminder notifications: (which of the following options do you like most?)
   1. Content of notifications
   2. Time and frequency
   3. Sound
   4. Repeated notifications if patients slide the message but don’t select Yes/No for taking pills.
2. Motivational messages: (which of the following options do you like most?)
   1. Content of messages
   2. Only present on the message tab or also alert by a push notification?
   3. Message only or with a chart? (eg. pie chart)
   4. Ability to export and share
3. Side-effect self-management:
   1. Would you want to receive a notification message recommending you to go to the app for more side-effects’ information?
   2. Would you want to receive a separate message reporting your side-effects’ status in the last 7 (?) weeks?
4. Adherence graphs:
   1. Will you likely to access the web portal to see your performance and other information?
   2. Graphs’ presentations (Which of the following options do you like most?)
5. Drugs’ cautions:
   1. Would you want to have information about drugs’ cautions in your Profile tab?
6. Presentations of the Tabs (which of the following options do you like most?)

Vertical, Horizontal

1. Other options
   1. Would you want to receive an alert for an upcoming phone consultation appointment?
   2. Would you want to have a feature on the app to add your family carer?
   3. Would you want to have a feature on the app to record and check the prescription stock/ refill?
   4. Would you want to receive an alert for an upcoming nurse’s/pharmacist’s phone consultation appointment?
