# Supplementary material for: “Take the tablet or don’t take the tablet?”—A qualitative study of patients’ experiences of self-administering anti-cancer medications related to adherence and managing side effects
Source: Support Care Cancer. 2023 Nov 7;31(12):680. doi: 10.1007/s00520-023-08122-6 (PMC10630231; doi:10.1007/s00520-023-08122-6)
Supplement: Supplementary file 2 — Supplementary file2 (DOCX 20 KB) [file 520_2023_8122_MOESM2_ESM.docx]

**Journal of Supportive Care in Cancer**

**Research**

“Take the tablet or don’t take the tablet?”–A qualitative study of patients’ experiences of self-administering anti-cancer medications related to adherence and managing side-effects

Appendix 2. Themes, categories, and sub-categories

**Theme 1-** **Varied factors affect patient motivation of medication adherence (MA)**

- 1. ***Cancer-related physical reactions and fears***
     1. People with cancer experience cognitive or psychological issues from diagnosis and new treatment
     2. Patients need the treatment for their urgent physical needs
     3. People with cancer may doubt the need for therapy when feeling OK
     4. Adverse cancer effects outweigh medicine side-effects, motivating MA
  2. ***Cancer literacy and beliefs***
     1. People with cancer aware of the importance of MA
     2. People with cancer can have beliefs about the disease
     3. Beliefs about the need for treatment
     4. Beliefs that being positive, determined, having a good quality of life and (or) fearful of medication non-adherent consequences are helpful in MA
     5. Religious belief can also motivate patients
  3. ***Healthcare professionals*** (***HCPs) communication***
     1. Their trust in HCPs motivates MA
     2. HCP's communication skills can influence how patients comply with the treatment
  4. ***Family and friends’ support networks can help disease management and MA***
     1. People with cancer can need and (or) value support from their network, such as family or general practitioners, for optimal medication adherence (MA) and well-being

**Theme 2-** **When motivated, MA and side-effect management strategies can develop over time.**

- 1. ***Developing personal strategies to support MA***
     1. The medication-taking habit can be self-initiated or developed when in the hospital
     2. People with cancer can adjust MA timing and intake to cope with daily life
     3. People with cancer can develop a personal strategy to ensure they don't double-dose
     4. MA can develop over time
     5. Strong mindset about the benefits and risks of taking medicine with side-effects
     6. Some people with cancer can occasionally, non-intentionally miss medications
  2. ***Managing side-effects through various strategies***
     1. Monitoring own body
     2. Self-experiment
     3. Self-medication
     4. Search for information online
     5. Consult healthcare professionals
     6. Use online sources of information and peer support
     7. Side-effects can be altered and managed over time

**Theme 3- Further HCP support needed while managing MA and side-effects**

#### **Need for regular follow-ups from consistent HCPs to support MA**

- - 1. Need to be connected with the care team
    2. Need to be able to contact an HCP when needed
    3. Can need to have a fixed HCP to follow up patients over time
    4. Inconsistent instructions from HCPs can cause patients to be frustrated
    5. Can need to feel more respected and fairly treated by HCPs
    6. Need responsive HCPs

#### **HCPs ongoing information, monitoring, and support could encourage MA**

- - 1. Many patients need prompts to remember to take drugs
    2. Most need ongoing medical personalised face-to-face or phone consults
    3. Need of information about the importance of MA, diseases and treatments, side-effects and management options, encouragement, and when to contact doctors for further levels of detail
    4. Can need specific questions from HCPs about MA and reasons for medication non-adherence, and options to manage
    5. Tailored follow-up contacts from HCPs can help to promote MA and people with cancer feeling of being valued, including those lacking confidence to contact HCPs
    6. People with cancer suggested a range of HCP follow up contact times in the first couple of weeks after starting treatment, from one to three times per week
